# A Review of the Management of Cholelithiasis at Buraydah Central Hospital in the Qassim Region, Saudi Arabia: A Cross-Sectional Study

**DOI:** 10.7759/cureus.50846

**Published:** 2023-12-20

**Authors:** Bandar Mutiri, Amarachukwu Chiduziem Etonyeaku, Mayar Aloufi, Jolan S Alsaud

**Affiliations:** 1 General Surgery, Buraydah Central Hospital, Buraydah, SAU; 2 Surgery, Buraydah Central Hospital, Buraydah, SAU; 3 Surgery, Obafemi Awolowo University, Ile-Ife, NGA; 4 Medicine, Qassim University, Buraydah, SAU

**Keywords:** stone in gallbladder, cholelithiasis, qassim region, gallbladder stone disease, gallstone disease

## Abstract

Cholelithiasis is the most common cause of gastroenterological hospitalization. Given this significant risk, perfectly managing cholelithiasis is crucial to reduce hospitalization. Unfortunately, we have not found a study on a review of the management of cholelithiasis in Saudi Arabia. Therefore, we aim to evaluate cholelithiasis concerning demographic features, presentation symptoms, predisposing risk factors, laboratory features, complications, and outcomes in the Qassim region. This cross-sectional study of all patients with a radiological diagnosis of gallstones, whether symptomatic or not, was diagnostic in 2022. The researchers fielded a preformed data collection sheet for each patient from the hospital system. Data obtained were entered into a spreadsheet and analyzed using SPSS Statistics version 23.0 (IBM Corp. Released 2015. IBM SPSS Statistics for Windows, Version 23.0. Armonk, NY: IBM Corp.). A total of 526 patients were included in the study. Most patients (116, 22.1%), were aged 38-42. The finding also indicated that most patients were females (397, 75.5%), while males were only 129 (24.5%). The study also found that the difference between women and men presenting complaints of fat intolerance and right shoulder pain was statistically significant. Women were 100 (84%) and men were 19 (16%) for fat intolerance, while for right shoulder pain, women were 50 (89.3%) and men were six (10.7%). The p-values were 0.014 and 0.011, respectively. Further, there was a statistically significant difference in terms of the complaints presented by the patients between the complicated and non-complicated cases of abdominal pain (p=0.001), nausea (p=0.001), vomiting (p=0.001), change in urine and stool color (p=0.001), and right shoulder pain (p=0.001), among other complaints (p=0.001). The study concludes that most patients affected by cholelithiasis are individuals in the middle age group, with women being more affected by the disease than men. Further, among the individuals affected by the disease, the majority of them were overweight and obese. On the other hand, the study concludes that the majority of patients who suffer more from cholelithiasis are affected mainly by other associated diseases such as hypertension, hypothyroidism, and diabetes mellitus. In conclusion, many factors may contribute to gallstone formation and the outcome of the disease and surgery. Therefore, the study recommends that health workers offer tailored education, especially targeting the highlighted factors found in this study, to create awareness of disease control measures in the general population. Also, keep in mind these factors when dealing with patients who complain of abdominal pain.

## Introduction

Cholelithiasis, otherwise called gallstone disease, is a disease characterized by the formation of stone(s) in the gallbladder from the aggregation of a mixture of water, cholesterol, bilirubin, and bile [1]. Cholesterol is derived from food, while bilirubin and bile are derived from the breakdown of the haem molecule in red blood cells. Usually, an equilibrium exists between these constituents, which are then maintained in a soluble state. However, disruptions of this equilibrium lead to super-saturation and precipitation of calcium hydrogen bilirubinate, the nucleation and polymerization of bile pigments, and the deposition of inorganic salts like calcium carbonate and phosphate. Gallstones can lead to a wide variety of clinical conditions, including acute cholecystitis, chronic cholecystitis, obstructive jaundice, and acute pancreatitis [2]. The majority of cases are asymptomatic and discovered incidentally, and it has been reported that approximately 20% of individuals experience symptomatic gallstone disease [3].

In a study in Riyadh in 2017, 8.6% of the study population had gallstone disease. They found out that the risk factors associated with gallstones in Saudi Arabia were aging, female sex, weight gain, diabetes mellitus, liver functional enzymes, and lipids [4]. With a gallstone prevalence rate of 11.7%, Abu-Eshy et al. reported that female gender, previous complaints of pancreatitis, and previous family cases of gallstone were strongly associated with gallstone disease, while age, blood pressure, coffee and cigarette intake, excess weight, diabetes, total pregnancies, and the use of oral contraceptives were to a lesser extent associated with the disease [5]. In another study to determine the factors contributing to the development of chronic cholecystitis in the senile population of the Kingdom of Saudi Arabia, the prevalence of the disease was about 7%. Similar to other studies, this study also reveals disease associations with age, gender, diabetes mellitus, and body mass index. This study further suggests that thyroid dysfunction and high blood pressure are associated with chronic cholecystitis [6].

*Helicobacter pylori* (*H. pylori*) causes infection in the mucosal layer of the stomach and gallbladder, and it is strongly associated with the formation of stones in the gallbladder. Guraya et al. [7], in Saudi Arabia, reported that *H. pylori* might be a potential risk factor for cholelithiasis. It has also been suggested that treatment of *H. pylori* infection is greatly needed to avoid the formation of stones in the gall bladder [2].

Previous studies had noted the relatively increased risk for gallstones among patients who had bariatric surgery and inferred that bariatric surgery contributes to excessive and rapid weight loss, which could be a significant risk factor for the development of cholelithiasis [8-10]. This has led to recommendations for using prophylactic bile acids, like ursodeoxycholic acid, in the early months after surgery to avoid cholecystitis and gallstone formation [11-13].

Reda et al. observed seasonal variation in the prevalence of acute cholecystitis among 161 patients, with higher rates in the summer (38%) than in the winter (22%) or autumn (18%) periods [14]. Moreover, Cariati posited that increasing age was a significant risk factor for both women and men [15]. Khazraei et al. reported that women have a higher prevalence of gallbladder stones than men [16]. The increase in prevalence of gallstones among women is thought to be more frequent due to obesity, which is higher in them [17]. In a case-control study, Ahmed et al. opined that genetic and environmental factors, like industrialization, cause obesity and fertility problems in women and may lead to gallstone development [18]. Besides obesity, other factors implicated in the etiopathogenesis of gallstones include female, 40, fertile, fatty, and fair (the so-called 5 "Fs" of gallstone disease), family history, and poor eating habits [19]. Finally, a report from China indicated that an active lifestyle, reduced weight gain, and less use of spices and fried foods would be beneficial in decreasing the prevalence of gallstone formation [20].

Even though cholelithiasis is the most common cause of gastroenterological hospitalization [21], we have not found a study on a review of the management of cholelithiasis in Saudi Arabia. Therefore, we aim to evaluate cholelithiasis concerning demographic features, presentation symptoms, predisposing risk factors, laboratory features, complications, and outcomes in the Qassim Region, Saudi Arabia.

## Materials and methods

This cross-sectional study was conducted at Buraydah Central Hospital, in the Qassim Region, Saudi Arabia. All patients diagnosed with cholelithiasis in 2022 were included in the study. The selection of samples was made by convenience sampling. The inclusion criteria were all patients reviewed and managed for gallstone disease with a radiological confirmation of stone or post-operative presence of stone in the gall bladder. The exclusion criteria were those who had uncompleted files. The sample size was calculated using Cochran's formula, with an expected prevalence of 50%, a confidence interval (CI) of 0.95, and a margin of error of 5% [22]. Consequently, a minimal sample size of 384 patients is needed to achieve the required CI. Our study includes a total of 526 participants to ensure accuracy.

The retrospective chart review of patients with cholelithiasis who were seen at Buraydah Central Hospital in 2022 was performed. Data were obtained from hospital medical files. We extracted data on age, gender, height, weight, comorbidity, presentation symptoms, previous surgery, lab result, type of surgery, other intervention if any, complication, and duration of hospital stay. The needed data were entered in a Google form (Google LLC, California, USA) and accessed by the researcher only to ensure data safety. Ethical approval was obtained from the Qassim Research Ethics Committee (approval no. 607/45/5833).

SPSS Statistics version 23.0 (IBM Corp. Released 2015. IBM SPSS Statistics for Windows, Version 23.0. Armonk, NY: IBM Corp.) was used to analyze the data. Furthermore, categorical data were used for descriptive statistics and were given as numbers, percentages, and frequencies, whereas continuous data were shown as mean, mode, and standard deviation. We utilized t-tests and chi-square to evaluate the significance of the relationship between the variables. P-values less than 0.05 were considered statistically significant.

## Results

Table 1 shows that a total of 526 respondents participated in the study, whereby most of the participants (116, 22.1%) were aged 38-42 years. In terms of gender, most of the respondents were women (397, 75.5%), while men were only 129 (24.5%). Regarding the BMI category, the highest number (207, 39.3%) were overweight, followed by obese (204, 38.8%), and then normal with 110 (20.9%), while only five (1%) were underweight. Additionally, comorbidities were high in patients with hypertension (44, 8.3%), diabetes mellitus (42, 8%), and hypothyroidism (33, 6.3%). Regarding complaints, a large number of the participants reported abdominal pain (388, 35.0%), vomiting (178, 16.0%), nausea (143, 12.9%), and fat intolerance (119, 10.7%). Further, most of the participants (201, 38.2%) had recurrent symptoms, while 156 (29.7%) had the symptoms for the first time. In terms of lab results, 454 (86.3%) were normal for WBC, 422 (80.2%) were normal for RBC, 413 (78.52%) were normal for alanine transaminase (ALT), 444 (84.41%) were normal for aspartate aminotransferase (AST), and 458 (87.07%) were normal for alkaline phosphatase (ALP). More so, 228 (43.3%) and 183 (34.8%) of the participants indicated day case and emergency, respectively, as the major surgery methods. Further, most of the participants (520, 98.8%) saw laparoscopy as the major type of surgery. Nevertheless, most of the participants (33, 6.3%) indicated upper endoscopy as an additional intervention. Among the complications of the surgery, abdominal pain was the most prevalent (11, 2.1%). Finally, the average duration of the hospital stay days was 2.74 with a standard deviation of 2.79.

**Table 1:** General characteristics of the participants (N=526) The data has been represented as N, %, mean ± SD ^ Some patients have more than one comorbidity and presenting complaints BMI: body mass index, WBC: white blood cell, RBC: red blood cell, HGB: hemoglobin, ALT: alanine transaminase, AST: aspartate aminotransferase, ALP: aspartate aminotransferase, MRCP: magnetic resonance cholangiopancreatography, ERCP: endoscopic retrograde cholangiopancreatography

Characteristics	Frequency (n)	Proportions (%)
Age group (years)		
18-22	28	5.3%
23-27	50	9.5%
28-32	77	14.6%
33-37	73	13.9%
38-42	116	22.1%
43-47	62	11.8%
48-52	43	8.2%
53-57	33	6.3%
58-62	18	3.4%
63-67	9	1.7%
68-72	9	1.7%
73-77	6	1.1%
78-82	1	0.2%
83-87	1	0.2%
Gender		
Males	129	24.5%
Females	397	75.5%
BMI category		
Underweight	5	1.0%
Normal	110	20.9%
Overweight	207	39.3%
Obesity	204	38.8%
Comorbidity^		
Non	363	69.0%
Diabetes mellitus	42	8.0%
Hypertension	44	8.3%
Hypothyroidism	33	6.3%
Dyslipidemia	5	1.0%
Kidney disease	5	1.0%
Pulmonary disease	21	4.0%
Cardiac disease	4	0.7%
Others	9	1.7%
Presenting complaints^		
Non	134	12.1%
Abdominal pain	388	35.0%
Nausea	143	12.9%
Vomiting	178	16.0%
Fat intolerance	119	10.7%
Change in urine or stool color	35	3.2%
Right shoulder pain	56	5.0%
Other	57	5.1%
Presentation of symptoms		
Recurrent	201	38.2%
First time	156	29.7%
No symptoms/miss	169	32.1%
Associated factors		
Non	438	83.3%
Lactation	7	1.3%
Postpartum	15	2.9%
Previous cesarean section	16	3.0%
Appendectomy	9	1.7%
Bariatric surgery	41	7.8%
Lab result of WBC		
Less than normal	53	10.1%
Normal	454	86.3%
More than the normal	19	3.6%
Lab result of RBC		
Less than normal	11	2.1%
Normal	422	80.2%
More than the normal	93	17.7%
Lab result of HGB		
Less than normal	47	8.9%
Normal	359	68.3%
More than the normal	120	22.8%
Lab result of ALT		
Normal	413	78.52%
More than the normal	96	18.25%
Missing data	17	3.23%
Lab result of AST		
Normal	444	84.41%
More than the normal	77	14.64%
Missing data	5	0.95%
Lab result of ALP		
Less than normal	458	87.07%
Normal	32	6.08%
More than the normal	8	1.53%
Missing data	28	5.32%
How the surgery done		
Elective	91	17.3%
Day case	228	43.3%
Emergency	183	34.8%
Urgent	24	4.6%
Type of the surgery		
Laparoscopy	520	98.8%
Open	3	0.6%
Not done	2	0.4%
Exploratory laparotomy	1	0.2%
The other Intervention		
Upper endoscopy	1	0.2%
MRCP	33	6.3%
ERCP	10	1.9%
MRCP and ERCP	17	3.2%
Hernia repair	10	1.9%
Appendectomy	1	0.2%
Excision of the umbilical mass	1	0.2%
Non	453	86.1%
Complication		
Surgical site infection	3	0.6%
Non	503	95.5%
Post-cholecystectomy syndrome	2	0.4%
Itching at the operation site	1	0.2%
Hematoma	2	0.4%
Dysuria	1	0.2%
Chest pain	1	0.2%
Cardiogenic shock	1	0.2%
Abdominal pain	11	2.1%
Obstruction of bile duct after the surgery	1	0.2%
Duration of the hospital days	2.74 ± 2.79	

**Figure 1:**
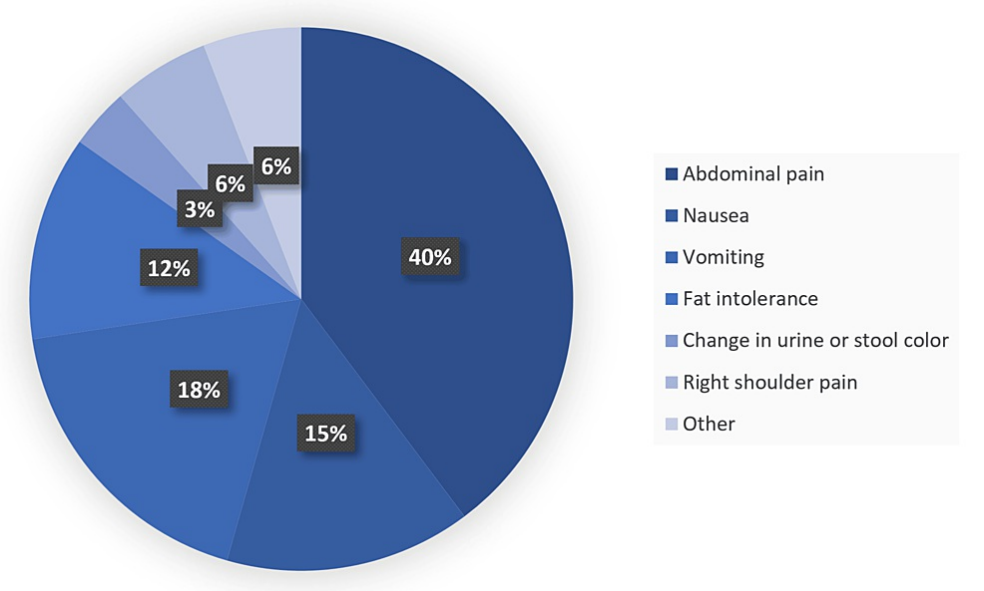
Presenting complaints

**Figure 2:**
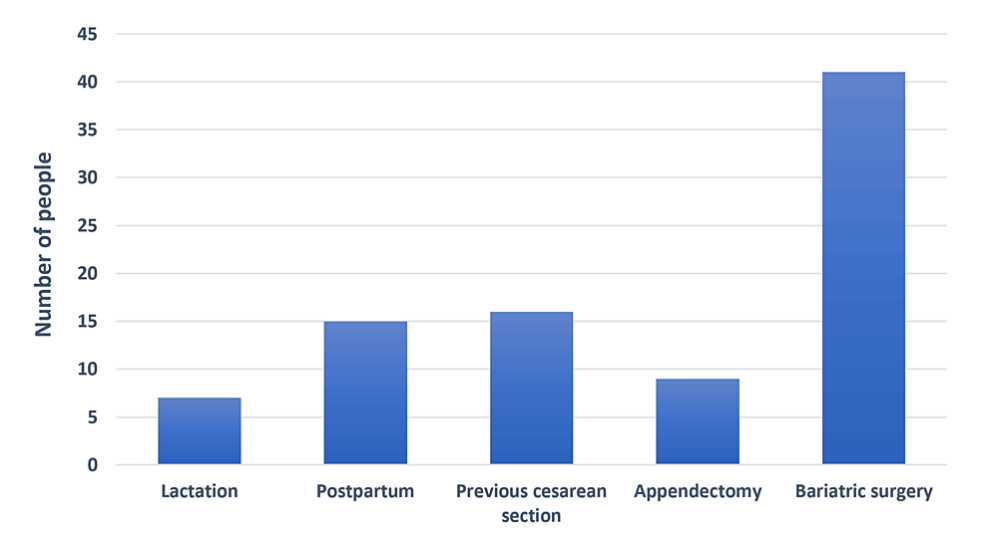
Associated factors of cholelithiasis

**Figure 3:**
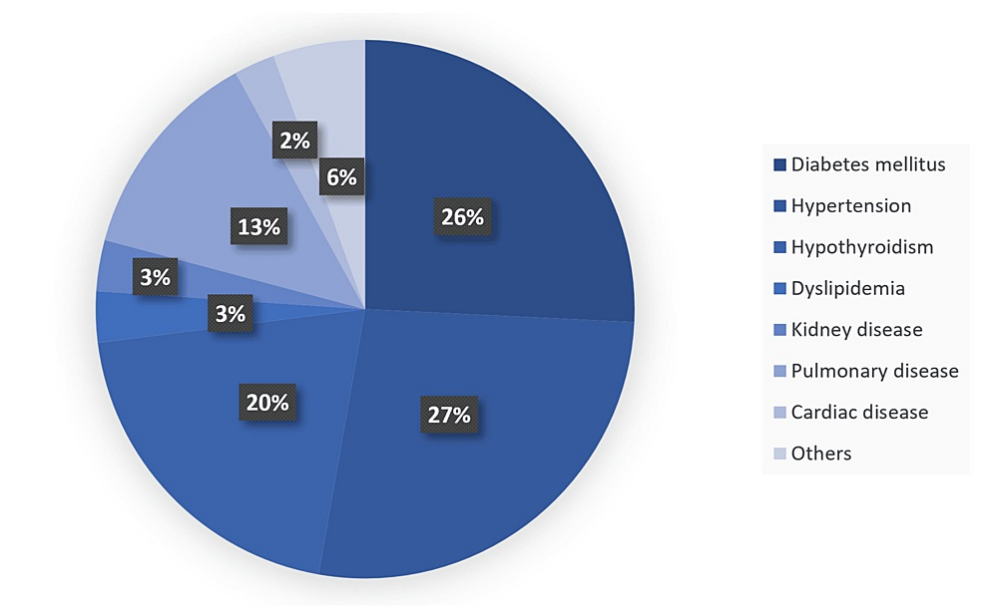
Participants' comorbidities

A majority of patients reported abdominal pain, followed by vomiting, nausea, and fat intolerance, respectively. Changes in urine or stool color resulted in the lowest reported symptoms (Figure 1). Bariatric surgery was the most common factor associated with 41 people. Other factors with a lower frequency included previous cesarean section (16 people), postpartum (15 people), appendectomy (nine people), and lactation (seven people) (Figure 2). Among comorbidities, hypertension ranked highest, followed by diabetes, hypothyroidism, and pulmonary disease, respectively. The lowest reported comorbidity was cardiac disease (Figure 3).

Table 2 shows that the difference between female and male participants presenting complaints of fat intolerance and right shoulder pain was statistically significant. Women were 100 (84%) and men were 19 (16%) for fat intolerance, while for right shoulder pain, women were 50 (89.3%) and men were six (10.7%). The p-values were 0.014 and 0.011, respectively. Additionally, the difference between women and men in terms of presentation of the symptoms was statistically significant: for recurrent (women (161, 80.1%), men (40, 19.9%)), first time (women (108, 69.2%), men (48, 30.8%)), p-value 0.007. The associated factors (lactation, postpartum, previous cesarean section (CS), appendectomy, and bariatric surgery) were shown to have a significant difference for women and men (p=0.007). More so, there exists a statistically significant difference between women's and men's lab results for WBC (p=0.009), RBC (p=0.001), and hemoglobin (HGB, p=0.001). Finally, a p-value of 0.007 indicated a statistically significant difference between female and male types of surgery.

**Table 2:** Association between gender and clinical characteristics (N=526) The data has been represented as N, % * A p-value of <0.05 is considered statistically significant ^ Some patients have more than one presenting complaints CS: cesarean section, WBC: white blood cell, RBC: red blood cell, HGB: hemoglobin, ALT: alanine transaminase, AST: aspartate aminotransferase, ALP: alkaline phosphatase, MRCP: magnetic resonance cholangiopancreatography, ERCP: endoscopic retrograde cholangiopancreatography

Characteristics	Total n (%)	Women n (%)	Men n (%)	p-value*
Presenting complaints ^				
Abdominal pain	388 (35.0%)	294 (75.8%)	94 (24.2%)	0.790
Nausea	143 (12.9%)	110 (76.9%)	33 (23.1%)	0.637
Vomiting	178 (16.0%)	130 (73%)	48 (27%)	0.392
Fat intolerance	119 (10.7%)	100 (84%)	19 (16%)	0.014
Change in urine or stool color	35 (3.2%)	25 (71.4%)	10 (28.6%)	0.565
Right shoulder pain	56 (5.0%)	50 (89.3%)	6 (10.7%)	0.011
Other	57 (5.1%)	40 (70.2%)	17 (29.8%)	0.325
Presentation of symptoms (N=357)				0.027
Recurrent	201 (56.3%)	161 (80.1%)	40 (19.9%)	
First time	156 (43.7%)	108 (69.2%)	48 (30.8%)	
Associated factors				0.007
Lactation	7 (1.3%)	7 (100%)	0 (0.00%)	
Postpartum	15 (2.9%)	15 (100%)	0 (0.0%)	
Previous CS	16 (3.0%)	16 (100%)	0 (0.0%)	
Appendectomy	9 (1.7%)	5 (55.6%)	4 (44.4%)	
Non	438 (83.3%)	321 (73.3%)	117 (26.7%)	
Bariatric surgery	41 (7.8%)	33 (80.5%)	12 (19.5%)	
Lab result (WBC)				0.009
Less than normal	53 (10.1%)	33 (62.3%)	20 (37.7%)	
Normal	454 (86.3%)	353 (77.8%)	101 (22.2%)	
More than the normal	19 (3.6%)	11 (57.9%)	8 (42.1%)	
Lab result (RBC)				0.001
Less than normal	11 (2.1%)	10 (90.9%)	4 (9.1%)	
Normal	422 (80.2%)	359 (85.1%)	63 (14.9%)	
More than the normal	93 (17.7%)	28 (30.1%)	65 (69.9%)	
Lab result (HGB)				0.001
Less than normal	47 (8.9%)	47 (100%)	0 (0.0%)	
Normal	359 (68.3%)	324 (90.5%)	34 (9.5%)	
More than the normal	120 (22.8%)	25 (20.8%)	65 (79.2%)	
Lab result (ALT) (N=509)				0.073
Normal	413 (81.1%)	316 (76.5%)	97 (23.5%)	
More than the normal	96 (18.9%)	65 (67.7%)	31 (32.3%)	
Lab result (AST) (N=521)				0.158
Normal	444 (85.2%)	339 (76.4%)	105 (23.6%)	
More than the normal	77 (14.8%)	53 (68.8%)	24 (31.2%)	
Lab result (ALP) (N=498)				0.229
Less than normal	458 (92%)	347 (75.8%)	111 (24.2%)	
Normal	32 (6.4%)	25 (78.1%)	7 (21.9%)	
More than the normal	8 (1.6%)	4 (50%)	4 (50%)	
How the surgery done				0.098
Elective	91 (17.3%)	69 (75.8%)	22 (24.2%)	
Day case	228 (43.3%)	183 (80.3%)	45 (19.7%)	
Emergency	183 (34.8%)	129 (70.5%)	54 (29.5%)	
Urgent	24 (4.6%)	16 (66.7%)	8 (33.3%)	
Type of the surgery				0.007
Laparoscopy	520 (98.8%)	396 (76.2%)	124 (23.8%)	
Open	3 (0.6%)	1 (33.3%)	2 (66.7%)	
Not done	2 (0.4%)	0 (0.0%)	2 (100%)	
Exploratory laparotomy	1 (0.2%)	0 (0.0%)	1 (100%)	
Other intervention (N=73)				0.767
Upper endoscopy	1 (0.2%)	0 (0.0%)	1 (100%)	
MRCP	33 (6.3%)	22 (66.7%)	11 (33.3%)	
ERCP	10 (1.9%)	7 (70%)	3 (30%)	
MRCP and ERCP	17 (3.2%)	13 (76.5%)	4 (23.5%)	
Hernia repair	10 (1.9%)	7 (70%)	3 (30%)	
Appendectomy	1 (0.2%)	1 (100%)	0 (0.0%)	
Excision of the umbilical mass	1 (0.2%)	1 (100%)	0 (0.0%)	
Complication				0.527
Surgical site infection	3 (0.6%)	2 (66.7%)	1 (33.3%)	
Non	503 (95.5%)	379 (75.3%)	124 (24.7%)	
Post-cholecystectomy syndrome	2 (0.4%)	2 (100%)	0 (0.0%)	
Itching at the operation site	1 (0.2%)	1 (100%)	0 (0.0%)	
Hematoma	2 (0.4%)	2 (100%)	0 (0.0%)	
Dysuria	1 (0.2%)	1 (100%)	0 (0.0%)	
Chest pain	1 (0.2%)	1 (100%)	0 (0.0%)	
Cardiogenic shock	1 (0.2%)	0 (0.0%)	1 (100%)	
Abdominal pain	11 (2.1%)	8 (72.7%)	3 (27.3%)	
Obstruction of bile duct after the surgery	1 (0.2%)	1 (100%)	0 (0.0%)	

Table 3 shows that the difference between the BMI categories of the participants presenting complaints of fat intolerance and changes in urine or stool color was statistically significant. The most prevalent was overweight (50, 42%) and obesity (19, 33.6%) for fat intolerance, while for changes in urine or stool color, overweight was 22 (62.9%) and obesity was eight (22.9%). The p-values were 0.014 and 0.011, respectively. Further, the associated factors (lactation, postpartum, previous CS, appendectomy, and bariatric surgery) were shown to have a significant difference in the BMI categories of the participants (p=0.001). In addition, a p-value of 0.013 indicates a statistically significant difference between the method of surgery and the BMI category.

**Table 3:** Association between BMI category and clinical characteristics (N=526) The data has been represented as N, % * A p-value of <0.05 is considered statistically significant ^ Some patients have more than one presenting complaints CS: cesarean section, WBC: white blood cell, RBC: red blood cell, HGB: hemoglobin, ALT: alanine transaminase, AST: aspartate aminotransferase, ALP: alkaline phosphatase, MRCP: magnetic resonance cholangiopancreatography, ERCP: endoscopic retrograde cholangiopancreatography

Characteristics	Underweight n (%)	Normal n (%)	Overweight n (%)	Obesity n (%)	p-value*
Presenting complaints ^					
Non	0 (0.0%)	1 (12.5%)	3 (37.5%)	4 (50%)	0.356
Abdominal pain	4 (1.0%)	85 (21.9%)	159 (41.0%)	140 (36.1%)	0.206
Nausea	2 (1.3%)	30 (21.0%)	57 (39.9%)	54 (37.8%)	0.923
Vomiting	3 (1.7%)	49 (27.5%)	62 (34.8%)	64 (36%)	0.028
Fat intolerance	4 (3.4%)	25 (21.0%)	50 (42.0%)	40 (33.6%)	0.013
Change in urine or stool color	2 (5.6%)	3 (8.6%)	22 (62.9%)	8 (22.9%)	0.001
Right shoulder pain	1 (1.8%)	8 (14.3%)	18 (32.1%)	29 (51.8%)	0.149
Other	2 (3.5%)	12 (21.1%)	21 (36.8%)	22 (38.6%)	0.212
Presentation of symptoms (N=357)					0.361
Recurrent	3 (1.5%)	45 (22.4%)	85 (42.3%)	68 (33.8%)	
First time	2 (1.3%)	33 (21.1%)	65 (41.7%)	56 (35.9%)	
Associated factors					0.001
Lactation	0 (0.0%)	1 (14.3%)	2(28.6%)	4 (57.1%)	
Postpartum	2 (13.3%)	3 (20%)	4 (26.7%)	6 (40%)	
Previous CS	0 (0.0%)	5 (31.2%)	4 (25.0%)	7 (43.8%)	
Appendectomy	0 (0.0%)	5 (55.6%)	1 (11.1%)	3 (33.3%)	
Non	3 (0.7%)	85 (19.4%)	184 (42%)	166 (37.9%)	
Bariatric surgery	0 (0.0%)	11 (26.8%)	12 (29.3%)	18 (43.9%)	
Lab result (WBC)					0.935
Less than normal	1 (1.9%)	13 (24.6%)	20 (37.7%)	19 (35.8%)	
Normal	4 (0.9%)	94 (20.7%)	180 (39.6%)	176 (38.8%)	
More than the normal	0 (0.0%)	3 (15.8%)	7 (36.8%)	9 (47.4%)	
Lab result (RBC)					0.681
Less than normal	0 (0.0%)	4 (36.4%)	3 (27.2%)	4 (36.4%)	
Normal	3 (0.7%)	87 (20.6%)	165 (39.1%)	167 (39.6%)	
More than the normal	2 (2.2%)	19 (20.5%)	39 (41.9%)	33 (35.4%)	
Lab result (HGB)					0.899
Less than normal	0 (0.0%)	10 (21.3%)	19 (40.4%)	18 (38.3%)	
Normal	6 (1.4%)	76 (21.2%)	141 (39.4%)	136 (38.0%)	
More than the normal	0 (0.0%)	24 (20.0%)	47 (39.2%)	49 (40.8%)	
Lab result (ALT) (N=509)					0.218
Normal	4 (1.0%)	80 (19.4%)	166 (40.2%)	163 (39.5%)	
More than the normal	0 (0.0%)	27 (28.1%)	36 (37.5%)	33 (34.4%)	
Lab result (AST) (N=521)					0.749
Normal	5 (1.2%)	92 (20.7%)	172 (38.7%)	175 (39.4%)	
More than the normal	0 (0.0%)	17 (22.1%)	32 (41.5%)	28 (36.4%)	
Lab result (ALP) (N=487)					0.420
Less than normal	3 (0.7%)	95 (23.4%)	177 (37.6%)	180 (38.3%)	
Normal	0 (0.0%)	6 (18.8%)	16 (50%)	10 (31.2%)	
More than the normal	0 (0.0%)	1 (12.5%)	6 (75%)	1 (12.5%)	
How the surgery done					0.013
Elective	2 (2.2%)	17 (18.7%)	38 (41.8%)	34 (37.3%)	
Day case	1 (0.3%)	57 (25.0%)	68 (28.0%)	102 (46.7%)	
Emergency	2 (1.1%)	30 (16.4%)	89 (48.6%)	62 (33.9%)	
Urgent	0 (0.0%)	6 (25%)	12 (50%)	6 (25%)	
Type of the surgery					0.436
Laparoscopy	5 (1.1%)	108 (20.7%)	207 (39.7%)	200 (38.5%)	
Open	0 (0.0%)	1 (33.3%)	0 (0.0%)	2 (66.7%)	
Not done	0 (0.0%)	0 (0.0%)	0 (0.0%)	2 (100%)	
Exploratory laparotomy	0 (0.0%)	1 (100%)	0 (0.0%)	0 (0.0%)	
The other Intervention (N= 73)					0.773
Upper endoscopy	0 (0.0%)	0 (0.0%)	1 (100%)	0 (0.0%)	
MRCP	0 (0.0%)	8 (24.2%)	11 (33.3%)	14 (42.5%)	
ERCP	0 (0.0%)	1 (10.0%)	8 (80.0%)	1 (10.0%)	
MRCP and ERCP	0 (0.0%)	4 (23.5%)	9 (53.0%)	4 (23.5%)	
Hernia repair	0 (0.0%)	1 (10.0%)	4 (40.0%)	5 (50.0%)	
Appendectomy	0 (0.0%)	0 (0.0%)	1 (100%)	0 (0.0%)	
Excision of the umbilical mass	0 (0.0%)	0 (0.0%)	0 (0.0%)	1 (100%)	
Complication					0.992
Surgical site infection	0 (0.0%)	0 (0.0%)	2 (33.3%)	3 (66.7%)	
Non	5 (1.0%)	104 (20.8%)	200 (39.9%)	194 (38.3%)	
Post-cholecystectomy syndrome	0 (0.0%)	1 (50%)	0 (0.0%)	1 (50%)	
Itching at the operation site	0 (0.0%)	0 (0.0%)	0 (0.0%)	1 (100%)	
Hematoma	0 (0.0%)	1 (50%)	0 (0.0%)	1 (50%)	
Dysuria	0 (0.0%)	0 (0.0%)	1 (100%)	0 (0.0%)	
Chest pain	0 (0.0%)	0 (0.0%)	0 (0.0%)	1 (100%)	
Cardiogenic shock	0 (0.0%)	0 (0.0%)	0 (0.0%)	1 (100%)	
Abdominal pain	0 (0.0%)	3 (27.2%)	4 (36.4%)	4 (36.4%)	
Obstruction of bile duct after the surgery	0 (0.0%)	0 (0.0%)	0 (0.0%)	1 (100%)	

Table 4 shows that the difference between the complicated and non-complicated was statistically significant hypothyroidism comorbidity (p=0.003). Further, there was a statistically significant difference in terms of the complaints presented by the participants between the complicated and non-complicated abdominal pain (p=0.001), nausea (p=0.001), vomiting (p=0.001), change in urine and stool color (p=0.001), right shoulder pain (p=0.001), and other complaints (p=0.001). Additionally, the difference between the complicated and non-complicated regarding the presentation of the symptoms was statistically significant (p=0.001). More so, there exists a statistically significant difference between the complicated and non-complicated lab results for WBC (p=0.001), ALT (p=0.001), AST (p=0.001), and ALP (p=0.001). There was also a statistically significant difference in how surgery was done, the type of surgery, and other interventions employed between the complicated and non-complicated. This was indicated by p-values of 0.001, 0.019, and 0.001, respectively.

**Table 4:** Association between complicated, non-complicated, and general characteristics (N=526) The data has been represented as N, % * A p-value of <0.05 is considered statistically significant ^ Some patients have more than one comorbidity and presenting complaints BMI: body mass index, CS: cesarean section, WBC: white blood cell, RBC: red blood cell, HGB: hemoglobin, ALT: alanine transaminase, AST: aspartate aminotransferase, ALP: alkaline phosphatase, MRCP: magnetic resonance cholangiopancreatography, ERCP: endoscopic retrograde cholangiopancreatography

Characteristics	Total N (%)	Non-complicated n (%)	Complicated n (%)	p-value*
Age group				0.314
18-22	28 (5.3%)	23 (82.1%)	5 (17.9%)	
23-27	50 (9.5%)	37 (74%)	13 (26%)	
28-32	77 (14.6%)	61 (79.2%)	16 (20.8%)	
33-37	73 (13.9%)	55 (75.3%)	18 (24.7%)	
38-42	116 (22.1%)	83 (71.6%)	33 (28.4%)	
43-47	62 (11.8%)	48 (77.4%)	14 (22.6%)	
48-52	43 (8.2%)	34 (79.1%)	9 (20.9%)	
53-57	33 (6.3%)	21 (65.7%)	12 (34.3%)	
58-62	18 (3.4%)	13 (72.2%)	5 (27.8%)	
63-67	9 (1.7%)	4 (44.4%)	5 (55.6%)	
68-72	9 (1.7%)	7 (77.8%)	2 (22.2%)	
73-77	6 (1.1%)	6 (100%)	0 (0.00%)	
78-82	1 (0.2%)	1 (100%)	0 (0.00%)	
83-87	1 (0.2%)	0 (0.00%)	1 (100%)	
Gender				0.085
Males	129 (24.5%)	89 (69.0%)	40 (31.0%)	
Females	397 (75.5%)	304 (76.6%)	96 (23.4%)	
BMI category				0.026
Underweight	5 (1.0%)	5 (100%)	0 (0.0%)	
Normal	110 (20.9%)	92 (82.2%)	18 (27.8%)	
Overweight	207 (39.3%)	144 (69.6%)	63 (30.4%)	
Obesity	204 (38.8%)	152 (74.5%)	52 (25.5%)	
Comorbidity^				
Diabetes mellitus	42 (25.6%)	33 (78.6%)	9 (21.4%)	0.711
Hypertension	44 (26.9%)	31 (70.5%)	13 (29.5%)	0.474
Hypothyroidism	33 (20.2%)	17 (51.5%)	16 (48.5%)	0.003
Dyslipidemia	5 (3.2%)	4 (80.0%)	1 (20.0%)	0.785
Kidney disease	5 (3.2%)	4 (80.0%)	1 (20.0%)	0.785
Pulmonary disease	21 (12.9%)	17 (81.0%)	4 (19.0%)	0.615
Cardiac disease	4 (2.5%)	1 (25.0%)	3 (75.0%)	0.052
Others	9 (5.5%)	7 (77.8%)	2 (22.2%)	0.831
Presenting complaints^				
Abdominal pain	388 (35.0%)	265 (68.3%)	123 (31.7%)	0.001
Nausea	143 (12.9%)	81 (56.6%)	62 (43.4%)	0.001
Vomiting	178 (16.0%)	97 (54.5%)	81 (45.5%)	0.001
Fat intolerance	119 (10.7%)	90 (75.6%)	29 (24.4%)	0.794
Change in urine or stool color	35 (3.2%)	9 (25.7%)	26 (74.3%)	0.001
Right shoulder pain	56 (5.0%)	30 (53.6%)	26 (46.4%)	0.001
Other	57 (5.1%)	31 (54.4%)	26 (45.6%)	0.001
Presentation of symptoms (N=357)				0.001
Recurrent	201 (56.3%)	163 (81.1%)	38 (18.9%)	
First time	156 (43.7%)	77 (49.4%)	79 (50.6%)	
Associated factors				0.273
Lactation	7 (1.3%)	7 (100%)	0 (0.00%)	
Postpartum	15 (2.9%)	9 (60%)	6 (40%)	
Previous CS	16 (3.0%)	12 (75%)	4 (25%)	
Appendectomy	9 (1.7%)	5 (55.6%)	4 (44.4%)	
Non	438 (83.3%)	331 (75.6%)	107 (24.4%)	
Bariatric surgery	41 (7.8%)	29 (70.7%)	12 (29.3%)	
Lab result (WBC)				0.001
Less than normal	53 (10.1%)	41 (77.4%)	12 (22.6%)	
Normal	454 (86.3%)	349 (76.9%)	105 (23.1%)	
More than the normal	19 (3.6%)	3 (15.8%)	16 (84.2%)	
Lab result (RBC)				0.146
Less than normal	11 (2.1%)	7 (63.6%)	4 (36.4%)	
Normal	422 (80.2%)	323 (76.5%)	99 (23.5%)	
More than the normal	93 (17.7%)	63 (67.7%)	30 (32.3%)	
Lab result (HGB)				0.193
Less than normal	47 (8.9%)	35 (74.5%)	12 (25.5%)	
Normal	359 (68.3%)	276 (77.1%)	83 (22.9%)	
More than the normal	120 (22.8%)	81 (67.5%)	39 (32.5%)	
Lab result (ALT) (N=509)				0.001
Normal	413 (81.1%)	342 (82.8%)	71 (17.2%)	
More than the normal	96 (18.9%)	37 (38.5%)	59 (61.5%)	
Lab result (AST) (N=521)				0.001
Normal	444 (85.2%)	369 (83.1%)	75 (16.9%)	
More than the normal	77 (14.8%)	19 (24.7%)	58 (75.3%)	
Lab result (ALP) (N=498)				0.001
Less than normal	458 (92%)	363 (79.3%)	95 (20.7%)	
Normal	32 (6.4%)	9 (28.1%)	23 (71.9%)	
More than the normal	8 (1.6%)	1 (12.5%)	54 (87.5%)	
How the surgery done				0.001
Elective	91 (17.3%)	83 (91.2%)	8 (8.8%)	
Day case	228 (43.3%)	222 (97.4%)	6 (2.6%)	
Emergency	183 (34.8%)	78 (42.6%)	105 (57.4%)	
Urgent	24 (4.6%)	10 (41.7%)	14 (58.3%)	
Type of the surgery				0.019
Laparoscopy	520 (98.8%)	390 (75%)	130 (25%)	
Open	3 (0.6%)	3 (100%)	0 (0.0%)	
Not done	2 (0.4%)	0 (0.0%)	2 (100%)	
Exploratory laparotomy	1 (0.2%)	0 (0.0%)	1 (100%)	
Other intervention				0.001
None	453 (86.1%)	372 (82.1%)	81 (17.9%)	
Upper endoscopy	1 (0.2%)	0 (0.0%)	1 (100%)	
MRCP	33 (6.3%)	9 (27.3%)	24 (72.7%)	
ERCP	10 (1.9%)	2 (20%)	8 (80%)	
MRCP and ERCP	17 (3.2%)	1 (5.9%)	16 (94.1%)	
Hernia repair	10 (1.9%)	7 (70%)	3 (30%)	
Appendectomy	1 (0.2%)	1 (100%)	0 (0.0%)	
Excision of umbilical mass	1 (0.2%)	1(100%)	0 (0.0%)	
Complication				0.527
Surgical site infection	3 (0.6%)	1(33.3%)	2 (66.7%)	
Non	503 (95.5%)	376 (75%)	125 (25%)	
Post-cholecystectomy syndrome	2 (0.4%)	1 (50%)	1 (50%)	
Itching at the operation site	1 (0.2%)	1 (100%)	0 (0.0%)	
Hematoma	2 (0.4%)	2 (100%)	0 (0.0%)	
Dysuria	1 (0.2%)	1 (100%)	0 (0.0%)	
Chest pain	1 (0.2%)	0 (0.0%)	0 (100%)	
Cardiogenic shock	1 (0.2%)	1 (100%)	0 (0.0%)	
Abdominal pain	11 (2.1%)	8 (72.7%)	3 (27.3%)	
Obstruction of bile duct after the surgery	1 (0.2%)	1 (100%)	0 (0.0%)	

## Discussion

The study reveals that a total of 526 respondents participated in the study. Most participants (116, 22.1%) were aged 38-42. The finding also indicates that most respondents were females (397, 75.5%), while males were only 129 (24.5%). Regarding the BMI category, the highest number of participants (207, 39.3%) were overweight, followed by obesity (204, 38.8%), and then normal with 110 (20.9%), while only five (1%) were underweight. Additionally, comorbidities were high in patients with hypertension (44, 8.3%), diabetes mellitus (42, 8%), and hypothyroidism (33, 6.3%). Regarding complaints, a large number of the participants reported abdominal pain (388, 35.0%), 178 (16.0%) vomiting, 143 (12.9%) nausea, and 119 (10.7%) fat intolerance. Further, most of the participants (201, 38.2%) had recurrent symptoms, while 156 (29.7%) had the symptoms for the first time. In terms of lab results, most of the respondents (454, 86.3%) were normal for WBC, 422 (80.2%) were normal for RBC, 413 (81.1%) were normal for ALT, 444 (85.2%) were normal for AST, and 458 (92%) were normal for ALP. More so, as sighted by most participants, the significant surgery methods were day case (228, 43.3%) and emergency (83, 34.8%). Further, most of the participants (520, 98.9%) undergo laparoscopy as the most significant type of surgery. Nevertheless, 33 participants (6.3%) indicated upper endoscopy as an additional intervention. Among the complications of the surgery, abdominal pain was the most prevalent (11, 2.1%). The average duration of the hospital stay days was 2.74, with a standard deviation of 2.79. These findings were similar to those acquired by Gehlot et al. in their study, which noted that the majority of the participants treated for cholelithiasis were characterized by overweight and obesity. At the same time, many were middle-aged individuals [23].

The study also found that the difference between female and male participants presenting complaints of fat intolerance and right shoulder pain was statistically significant. Women were 100 (84%), and men were 19 (16%) for fat intolerance, while for right shoulder pain, women were 50 (89.3%) and men were six (10.7%). The p-values were 0.014 and 0.011, respectively. Additionally, the difference between the women and men in terms of presentation of the symptoms was statistically significant for recurrent (women (161, 80.1%), men (40, 19.9%)), first time (women (108, 69.2%), men (48, 30.8%), p-value of 0.007. The associated factors (lactation, postpartum, previous CS, appendectomy, and bariatric surgery) were shown to have a significant difference for women and men (p=0.007). More so, there exists a statistically significant difference between women's and men's lab results for WBC (p=0.009), RBC (p=0.001), and HGB (p=0.001). Finally, a p-value of 0.007 indicated a statistically significant difference between female and male types of surgery. These findings are in line with the findings obtained by Tuna et al. in their study, which noted that a large number of patients characterized by cholelithiasis symptoms were female as compared to male patients, with most of them having recurrent symptoms [24].

More so, the findings reveal that the difference between the BMI categories of the participants presenting complaints of fat intolerance and changes in urine or stool color was statistically significant. The most prevalent was overweight (50, 42%) and obesity (19, 33.6%) for fat intolerance, while for change in urine or stool color, overweight was 22 (62.9%) and obesity was eight (22.9%). The p-values were 0.014 and 0.011, respectively. Further, the associated factors (lactation, postpartum, previous CS, appendectomy, and bariatric surgery) were shown to have a significant difference for the BMI categories of the participants (p=0.001). In addition, a p-value of 0.013 indicates a statistically significant difference between the surgery method and BMI category. These outcomes were similar to the outcomes obtained by Gökçe et al. in their study, which noted that the middle-aged group is more affected by cholelithiasis since the majority have no control over their eating habits, which is a contributing factor to the emergence of cholelithiasis [25].

Further, the study indicates that the difference between the complicated and non-complicated was statistically significant for hypothyroidism comorbidity (p=0.003). Further, there was a statistically significant difference in terms of the complaints presented by the participants between the complicated and non-complicated abdominal pain (p=0.001), nausea (p=0.001), vomiting (p=0.001), change in urine and stool color (p=0.001), right shoulder pain (p=0.001), and other complaints (p=0.001). Additionally, the difference between the complicated and non-complicated regarding the presentation of the symptoms was statistically significant (p=0.001). More so, there exists a statistically significant difference between the complicated and non-complicated lab results for WBC (p=0.001), AST (p=0.001), ALP (p=0.001), and ALT (p=0.001). Finally, the study findings reveal a statistically significant difference in how surgery was done, the type of surgery, and other interventions employed between the complicated and non-complicated. This was indicated by p-values of 0.001, 0.019, and 0.001, respectively. These findings agree with the findings acquired by Al-Saad et al. in their study, which noted that cholelithiasis patients suffering from other serious diseases, such as hypertension and hypothyroidism, have more severe symptoms of cholelithiasis than patients who are not affected by other diseases [26]. Also, according to the findings obtained by Deepak et al. in their study, comorbidity significantly influences the level of cholelithiasis management as it involves the management of other associated diseases, which sometimes makes it hard to manage the primary disease [27].

This study has several limitations, including a small sample size, which limits generalizability to a larger population. Data collected from the hospital system limited our ability to obtain additional information, such as family history and smoking. Additionally, *H. pylori* and other risk factors were not included in our study.

## Conclusions

The majority of the patients affected by cholelithiasis are individuals in the middle age group, with women being more affected by the disease as compared to men. Further, among the individuals affected by the disease, the majority of them fall under the categories of overweight and obese. On the other hand, the majority of patients who suffer more from cholelithiasis are affected mainly by other associated diseases such as hypertension, hypothyroidism, and diabetes mellitus, among others. Many factors may contribute to gallstone formation and the outcome of the disease and surgery. Therefore, the study recommends that health workers offer tailored education, especially targeting the highlighted factors found in this study, to create awareness of disease control measures in the general population. Also, keep in mind these factors when dealing with patients who complain of abdominal pain. The study also recommends future research be conducted evaluating the feeding of the affected group in order to establish the significant contributing factor to the number of cholelithiasis cases reported.

## References

[1] Reshetnyak VI (2012). Concept of the pathogenesis and treatment of cholelithiasis. World J Hepatol.

[2] Abayli B, Colakoglu S, Serin M (2005). Helicobacter pylori in the etiology of cholesterol gallstones. J Clin Gastroenterol.

[3] Stinton LM, Shaffer EA (2012). Epidemiology of gallbladder disease: cholelithiasis and cancer. Gut Liver.

[4] Alishi YA, Howaish FA, Alhamdan FA (2017). Prevalence and risk factors for gallstones among population in Riyadh city, KSA 2017. Egypt J Hosp Med.

[5] Abu-Eshy SA, Mahfouz AA, Badr A, El Gamal MN, Al-Shehri MY, Salati MI, Rabie ME (2007). Prevalence and risk factors of gallstone disease in a high altitude Saudi population. East Mediterr Health J.

[6] Alanazi M, Ali A, Alaleimi W (2018). Prevalence and determinant factors of chronic calculous cholecystitis among senile population Arar, KSA. Egypt J Hosp Med.

[7] Guraya SY, Ahmad AA, El-Ageery SM, Hemeg HA, Ozbak HA, Yousef K, Abdel-Aziz NA (2015). The correlation of Helicobacter Pylori with the development of cholelithiasis and cholecystitis: the results of a prospective clinical study in Saudi Arabia. Eur Rev Med Pharmacol Sci.

[8] Ibrahim A, Baiomy TA, Amin MF, Allam AS, Harb OA, Abdalla WM (2021). Incidence and risk factors for development of cholelithiasis after bariatric surgeries in Egypt
a prospective cohort study. Egypt J Cataract Refract Surg.

[9] Aldriweesh MA, Aljahdali GL, Shafaay EA (2020). The incidence and risk factors of cholelithiasis development after bariatric surgery in Saudi Arabia: a two-center retrospective cohort study. Front Surg.

[10] Jerais SA, Ahmad RM, Taleb MI (2018). Evaluation of the association of bariatric surgery with development of cholelithiasis. Egypt J Hosp Med.

[11] Machado FH, Castro Filho HF, Babadopulos RF, Rocha HA, Rocha JL, Moraes Filho MO (2019). Ursodeoxycholic acid in the prevention of gallstones in patients subjected to Roux-en-Y gastric bypass1. Acta Cir Bras.

[12] Lee SH, Jang DK, Yoo MW (2020). Efficacy and safety of ursodeoxycholic acid for the prevention of gallstone formation after gastrectomy in patients with gastric cancer: the Pegasus-D randomized clinical trial. JAMA Surg.

[13] Nabil TM, Nafady HA, Soliman SS, Hussein A, Mostafa KG (218). Cholelithiasis prophylaxis after bariatric procedures using ursodeoxycholic acid (UDCA). J Obes Weight Loss Ther.

[14] Reda AS, Aboulkhair LA, Almsarri MY (2015). The effect of seasonal variation on developing acute cholecystitis among adult patients in Jeddah, Saudi Arabia. Int J Health Sci.

[15] Cariati A (2015). Gallstone classification in western countries. Indian J Surg.

[16] Hosseini SV, Ayoub A, Rezaianzadeh A, Bananzadeh AM, Ghahramani L, Rahimikazerooni S, Khazraei H (2016). A survey on concomitant common bile duct stone and symptomatic gallstone and clinical values in Shiraz, Southern Iran. Adv Biomed Res.

[17] Dittrick GW, Thompson JS, Campos D, Bremers D, Sudan D (2005). Gallbladder pathology in morbid obesity. Obes Surg.

[18] Ahmed AF, El-Hassan OM, Mahmoud ME (1992). Risk factors for gallstone formation in young Saudi women: a case control study. Ann Saudi Med.

[19] Dash RN, Satpathy G (2018). USG evaluation of cholelthiasis in relation to 5fs mnemonics in addition to two other factors like family history and food. Adv Biomed Res.

[20] Zhu L, Aili A, Zhang C, Saiding A, Abudureyimu K (2014). Prevalence of and risk factors for gallstones in Uighur and Han Chinese. World J Gastroenterol.

[21] Gutt C, Schläfer S, Lammert F (2020). The treatment of gallstone disease. Dtsch Arztebl Int.

[22] (2023). OpenEpi. https://www.openepi.com/Menu/OE_Menu.htm.

[23] Gehlot CR, Sharma G, Mahala MK, Bhargava R, Malviya A (2020). Retrospective and prospective study on Cholelithiasis: various modes of management, their results, complications and histopathological changes in the gall bladder. Int Surg J.

[24] Tuna Kirsaclioglu C, Çuhacı Çakır B, Bayram G, Akbıyık F, Işık P, Tunç B (2016). Risk factors, complications and outcome of cholelithiasis in children: a retrospective, single-centre review. J Paediatr Child Health.

[25] Gökçe S, Yıldırım M, Erdoğan D (2014). A retrospective review of children with gallstone: single-center experience from Central Anatolia. Turk J Gastroenterol.

[26] Al-Saad MH, Alawadh AH, Al-Bagshi AH (2018). Surgical management of cholelithiasis. Egypt J Hosp Med.

[27] Deepak J, Agarwal P, Bagdi RK, Balagopal S, Madhu R, Balamourougane P (2009). Pediatric cholelithiasis and laparoscopic management: a review of twenty two cases. J Minim Access Surg.

